# An Integrated Fuzzy C-Means Method for Missing Data Imputation Using Taxi GPS Data

**DOI:** 10.3390/s20071992

**Published:** 2020-04-02

**Authors:** Junsheng Huang, Baohua Mao, Yun Bai, Tong Zhang, Changjun Miao

**Affiliations:** 1 School of Traffic and Transportation, Beijing Jiaotong University, Beijing 100044, China; 19114037@bjtu.edu.cn (J.H.); yunbai@bjtu.edu.cn (Y.B.); 16114195@bjtu.edu.cn (T.Z.); 2 Key Laboratory of Transport Industry of Big Data Application Technologies for Comprehensive Transport, Beijing Jiaotong University, Beijing 100044, China; 3 Integrated Transportation Research Centre of China, Beijing Jiaotong University, Beijing 100044, China; 4 Signal & Communication Research Institute, China Academy of Railway Sciences Corporation Limited, Beijing 100081, China; 0216008@stu.lzjtu.edu.cn

**Keywords:** Intelligent Transportation System, missing values imputation, fuzzy C-means, genetic algorithm

## Abstract

Various traffic-sensing technologies have been employed to facilitate traffic control. Due to certain factors, e.g., malfunctioning devices and artificial mistakes, missing values typically occur in the Intelligent Transportation System (ITS) sensing datasets, resulting in a decrease in the data quality. In this study, an integrated imputation algorithm based on fuzzy C-means (FCM) and the genetic algorithm (GA) is proposed to improve the accuracy of the estimated values. The GA is applied to optimize the parameter of the membership degree and the number of cluster centroids in the FCM model. An experimental test of the taxi global positioning system (GPS) data in Manhattan, New York City, is employed to demonstrate the effectiveness of the integrated imputation approach. Three evaluation criteria, the root mean squared error (RMSE), correlation coefficient (R), and relative accuracy (RA), are used to verify the experimental results. Under the ±5% and ±10% thresholds, the average RAs obtained by the integrated imputation method are 0.576 and 0.785, which remain the highest among different methods, indicating that the integrated imputation method outperforms the history imputation method and the conventional FCM method. On the other hand, the clustering imputation performance with the Euclidean distance is better than that with the Manhattan distance. Thus, our proposed integrated imputation method can be employed to estimate the missing values in the daily traffic management.

## 1. Introduction

**Figure 1:**
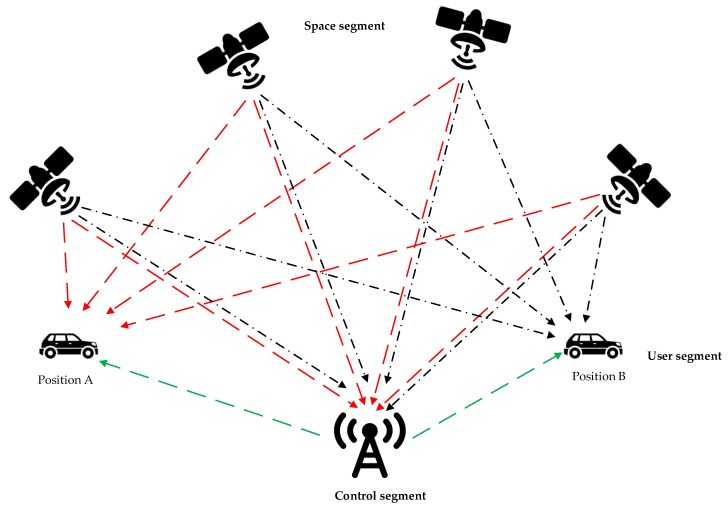
Working of GPS.

With the advent of the intelligent transportation era, various traffic sensors, including loop detectors, cameras, and GPS receivers, have been widely adopted to facilitate traffic control and management. Taking GPS as an example, as seen in Figure 1, it consists of three parts, including the space segment, the user segment, and the control segment. The user segment consists of GPS receivers and the user community. Currently, almost all taxis are required to be equipped with GPS receivers for location recording. Hence, a substantial amount of taxi mobile data from taxis is accurately recorded in the taxi GPS datasets. As seen in Table 1, the passengers’ pick-up and drop-off locations, dates, etc., are easily obtained as they are recorded in the taxi GPS datasets. 

Currently, many applications have been made by analyzing taxi GPS data. For example, by utilizing taxi trajectory data, passengers’ daily travel characteristics are mined [1]. Meanwhile, the real-time status of urban roads can be predicted as well [2]. Typically, the passengers’ mobile data generated by the GPS reflect the dynamic states of the transportation network. Specifically, the dynamic states are the outcomes of different demands of commuters and travelers. Thus, another interesting topic focuses on taxi demand distribution learning [3]. By analyzing taxi demand, intelligent taxi dispatch can be achieved as well [4].

However, due to the malfunctions of hardware or software, the issue of missing data arises frequently. Consequently, it poses a challenge to transportation planning and management [5], passenger behavior analysis, and demand forecasting [6,7]. Thus, it is crucial to deal with the missing data issue.

In general, there are various approaches that can be used to address the missing data. Ignoring, deleting, and zeroing are unsophisticated methods [8]. The common drawback of these methods is that valid information in the missing data is ignored. Furthermore, the lack of complete data would reduce the data quality and restrict data applications. Therefore, in order to obtain high-quality data, missing data should be generated by estimating effectively and efficiently [9].

In general, the imputation method mainly involves two types of approaches [10]. First, missing data are predicted by using the statistical method [11]. Specifically, some statistical features, including the mean and various indicators, are utilized to replace the missing values. Second, the missing values are estimated by machine learning techniques [12]. In particular, these techniques include fuzzy C-means (FCM) [13], support vector machine (SVM) [14], and random forest [15]. The core procedure is to generate several candidate values by using more than one model. Then, the missing data are replaced by the best candidate values, which are determined by some evaluation criteria.

Motivated by recent works in the literature [16], this paper aims to establish a novel imputation method integrating the matrix-based method, the fuzzy C-means (FCM) method, and the genetic algorithm (GA). (1) It should be noted that taxi demand volume data on weekdays or weekends in the taxi GPS datasets share periodic similarity spatiotemporally. Taking this into consideration, the matrix-based method is utilized to visualize this data pattern instead of the conventional vector-based method. (2) For the FCM method, it is widely used to deal with the clustering problem with incomplete data [17,18]. Specifically, the FCM method is capable of generating satisfactory estimation results by analyzing multiple dimensional datasets. However, these clustering results are sensitive to the parameter of the membership degree and the number of cluster centroids. Therefore, in our study, a GA is incorporated into the FCM to optimize the above two critical parameters.

The rest of this paper is organized as follows: Section 2 reviews the related studies in recent years. Section 3 presents the integrated methods for estimating missing data. Section 4 demonstrates an experimental test of taxi GPS data in Manhattan, New York City, in 2013, and provides comparisons and analyses of the results. Finally, Section 5 summarizes some findings and proposes future works.

## 2. Literature Review

In recent years, during the process of analyzing traffic data from various datasets, one problem that has caused extensive concern is the missing traffic value. Tian et al. [19] found that missing traffic flow data occurred frequently when they dealt with long short-term predictions of the traffic flow. Specifically, Chen et al. [20] pointed out that the missing data problem was common in floating sensing and crowdsourcing systems. Similarly, Ni et al. [21] found that raw traffic flow data accumulated by the Intelligent Transportation Systems (ITSs) were typically incomplete, which might render some datasets useless. Thus, to obtain missing values, different imputation methods should be proposed.

### 2.1. Deletion Method

Before presenting various imputation methods, one conventional method should be firstly reviewed. The deletion method is a time-saving and common choice when the cardinality of missing data is relatively small. As the name implies, the deletion method means missing data or missing variables are substituted by other data or variables. Due to its simplicity, this deletion method is extensively utilized in data preprocessing [22].

When the cardinality of missing data becomes larger, other methods that provide estimations of the missing data should be strongly recommended. Currently, with the aim to ensure that the estimated data are closer to the real data, other imputation methods that are based on the correlation between the missing data and the other existing data are being developed. In particular, these imputation methods include the regression imputation method [23,24,25,26], the k-nearest neighbor imputation (KNNI) method [27,28,29], the expectation maximization imputation (EMI) method [30], the knowledge-based method [31,32], and the fuzzy C-means method [33,34,35].

### 2.2. Regression Imputation Method

Regression imputation is a simple and common way of dealing with missing values. Chen et al. [36] firstly investigated how pairs of neighboring detectors behave, and then established a linear regression model to estimate the missing values. Boyles et al. [37] compared the performances of eleven approaches based on three types of missing data. The eleven approaches included a simple linear regression model, multiple linear regression, local and global regression, and historical imputation. The three types of missing data consisted of random losses data, continuous losses data, and systematic losses data. The imputation results indicated that the regression model was highly sensitive to the input data, and the estimated results obtained by the historical method were less accurate. Although the regression methods are easy to apply, traffic conditions vary constantly, and the estimation performance might be unreliable.

### 2.3. KNNI Method

Another imputation method is k-nearest neighbor imputation (KNNI). It should be noted that the k value in the KNNI means that the number of neighbors near each value is k. Batista et al. [38] utilized the Euclidean distance to find the predefined k value of records from the total dataset. They replaced the missing value with the mean value of its neighbors. Troyanskaya et al. [39] proposed the weight k-nearest neighbor imputation (WKNNI) to estimate the missing values, and the estimation results showed that the imputation performance of the WKNNI was more robust than that of the original KNNI. In general, if the dataset was insufficient, the KNNI could perform well. However, as the dataset became larger, the KNNI could be exceedingly time consuming because this method would find the k value of similar records from the whole dataset for each missing value.

### 2.4. EMI Method

Malan et al. [30] pointed out that the critical step in the expectation maximization imputation (EMI) was to develop the maximum likelihood estimation (MLE) [40,41]. Then, missing data were predicted by a known probability distribution of the MLE, and the iteration was terminated when the estimated data stopped changing. Therefore, this method is only applicable to data missing at random. Evidently, the main drawback of the EMI is the estimation of parameters through the MLE, and how to provide a reasonable assumption of the estimated parameters through the MLE is another interesting topic.

### 2.5. Knowledge-Based Method

Qi et al. [31] pointed out that existing imputation methods lack extra knowledge. In addition, the knowledge-based model was capable of capturing missing values with a public knowledge base [42]. Although the knowledge-based method can fill missing values with the help of human intelligence, several drawbacks also exist. One drawback is that the type of missing data is mismatched. Mismatching data would affect the accuracy of the estimated results. Another drawback is the absence of the potential knowledge of the missing values. This problem would also result in low accuracy in the imputation procedure.

### 2.6. Fuzzy C-Means Method

In addition, the clustering method is another type of imputation method. The common clustering method typically is comprised of K-means and fuzzy K-means. Amiri et al. [10] pointed out that the central problem in the K-means was the determination of the centroid positions of clusters. These centroid positions were iterated to update them according to the calculated distance and judgment conditions. Then, the missing value was replaced by the nearest neighbor based on its newest cluster [10]. Li et al. [43] utilized the fuzzy K-means method to estimate the missing values. The fuzziness meant that each instance did not belong to a cluster completely. The estimation results showed that a more robust clustering was obtained after numerical tests [43]. Theoretically, the main obstacle of applying the fuzzy K-means is how to determine the cluster number and the membership degree [44,45,46]. It should be noted that the selection of the cluster number and the determination of the membership degree cannot follow an artificial method. Thus, fuzzy K-means based on the hybrid algorithm merits further investigation.

## 3. Methodology

### 3.1. Research Framework

**Figure 2:**
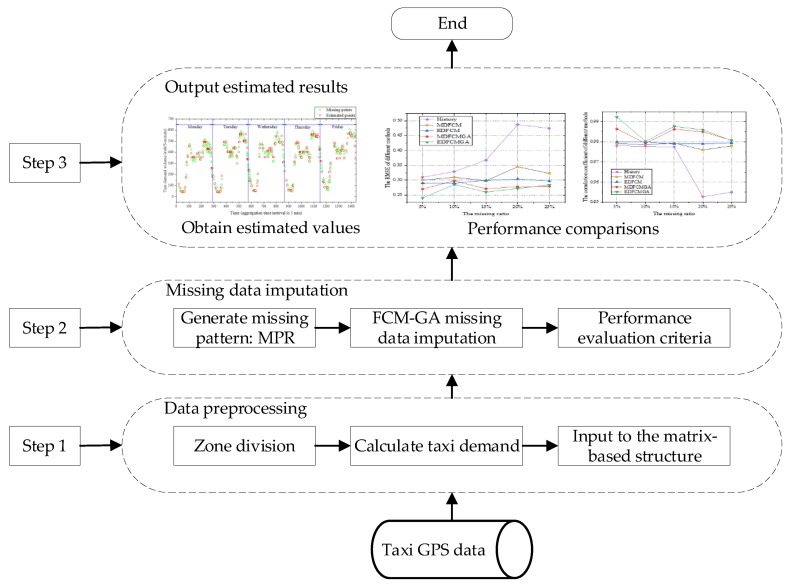
Workflow of the research framework. MPR means missing partially at random; FCM-GA means fuzzy C-means with genetic algorithm.

Before introducing the proposed methodology, the overall research framework is depicted as follows:

It can be seen from Figure 2 that the research framework contained three steps. In Step 1, the raw taxi GPS data are processed based on different passengers’ pick-up locations, in which taxi demands are generated. Then, taxi demands of specific zones on each weekday are input into the matrix-based structure. In Step 2, the values in the matrix-based structure are randomly deleted according to the specific missing pattern. Then, an imputation method is proposed to generate the estimated values, and several performance evaluation criteria are selected. In Step 3, the imputation results and comparison results are illustrated, respectively.

### 3.2. Matrix-Based Missing Data Description

From Qu et al. [47], three kinds of missing data are classified according to the missing data characteristics.

(1) Missing completely at random (MCR): missing values are independent of any other values;

(2) Missing partially at random (MPR): missing values have a relationship with other existing values, and the missing values could be estimated by other existing values;

(3) Missing not at random (MNR) or missing due to systematical errors [12]: missing data have a relationship not only with other missing values, but with other existing values, and cannot be estimated just by using other existing values.

**Figure 3:**
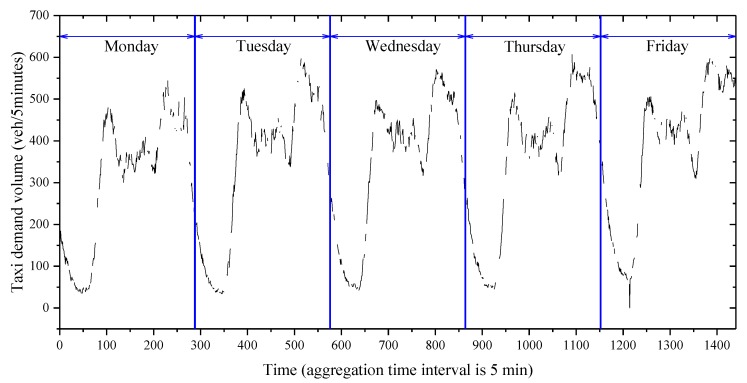
Incomplete data on weekdays based on a 5 min aggregation interval.

Thanks to the GPS technique, firstly, we calculated the number of pick-ups by taxi in the region of Manhattan, New York City, to represent the taxi demand volume. Secondly, due to the limitation of the taxi GPS datasets, we assumed that the type of missing data belonged to the MPR. Moreover, the observed taxi demand volume data from the taxi GPS datasets and missing data at a 25% missing ratio are shown in Table 2 and Figure 3.

The “question marks” in Table 2 represent the missing values. Specifically, the data filled in each column denote the same days of one week. We can see that if the collection interval was set as 5 min, the length of each column would be 288.

Schematically, as shown in Figure 3, the taxi demand volume data collected on each weekday usually had two peaks. Meanwhile, the height and the position of each peak shared similarities to some extent. These patterns fell into the category of data similarity. Theoretically, one of the mathematical methods to include the data similarity is the matrix-based structure. The advantage of the matrix-based method is that explicit topological information around the missing data is utilized; therefore, the data imputation accuracy is improved. The general form is represented by Equation (1).

### 3.3. Conventional Fuzzy C-Means Imputation Algorithm

The fuzzy C-means clustering algorithm is one of the most efficient clustering techniques [13]. Meanwhile, this technique is also capable of estimating missing values in the incomplete datasets. Before utilizing the FCM, several notations definition should be clearly given. Note that there are 288 rows in the matrix of Table 2, and each row has five columns. Let X represent the raw data matrix, and $X=\{x_{1};x_{2};\ldots ;x_{k};\ldots ;x_{n}\}$ is easily derived, where n is 288 in Table 2. Due to the p attributes in each row, $x_{k}=\{x_{1k},x_{2k},\ldots x_{jk},\ldots x_{pk}\}$ is also derived, where p is 5 in Table 2. The matrix-based structure is of the following form:

(1) $$X=\left[\begin{array}{ccc} x_{11} & \ldots & x_{p1}\\ \vdots & x_{jk} & \vdots \\ x_{1n} & \ldots & x_{pn} \end{array}\right]$$

Moreover, c denotes the number of clusters. Specifically, with respect to each cluster, 1≤i≤c, and let $y_{i}$ denote each cluster. Different from Tang et al. [12], in order to increase the estimation accuracy, each cluster $y_{i}$ also is comprised of p attributes, and each attribute denotes the cluster centroid. Thus, it is easily obtained that $y_{i}=\{y_{1i},y_{2i},\ldots y_{ji},\ldots y_{pi}\}$.

Different from the K-means clustering algorithm, one of the features of the fuzzy C-means algorithm is the membership degree $u(x_{k},y_{ji})$, which represents how close it is between $x_{k}$ and cluster centroid $y_{ji}$. The indicator used to distinguish the membership degree is the distance $d(x_{k},y_{ji})$. Note that when t in the Formula (3) is 1, the distance is the Manhattan distance; when t is 2, the distance is the Euclidean distance.

Due to the membership degree and the distance, the objective function of the FCM should be comprised of both simultaneously, which is shown in Formula (2). The distance between any point and the cluster centroid is calculated by Formula (3). The membership degree is calculated by Formula (4). The sum of the membership degree of each $x_{k}$ should be equal to one, which is shown in Formula (5). When the difference between the new membership degree $u{\left(x_{k},y_{ji}\right)}^{*}$ and the old membership degree $u(x_{k},y_{ji})$ is larger than the threshold ε, the new cluster centroid should be updated by Formula (6). When the optimal cluster centroids are finally obtained, the missing values can be obtained by Formula (7).

(2) $$\min\;J=\sum_{k=1}^{n}\sum_{i=1}^{c}\sum_{j=1}^{p}u{\left(x_{k},y_{ji}\right)}^{m}\cdot d(x_{k},y_{ji})$$

(3) $$d(x_{k},y_{ji})=\sum_{g=1}^{p}{\left(x_{gk}-y_{ji}\right)}^{1/t}$$

(4) $$u(x_{k},y_{ji})=1/[\sum_{a=1}^{c}{\left(\frac{d(x_{k},y_{ji})}{d(x_{k},y_{ja})}\right)}^{1/(m-1)}]$$

(5) $$\sum_{j=1}^{p}u(x_{k},y_{ji})=1$$

(6) $$y_{ji}=\frac{\sum_{k=1}^{n}\left[u{\left(x_{k},y_{ji}\right)}^{m}\cdot x_{jk}\right]}{\sum_{k=1}^{n}u{\left(x_{k},y_{ji}\right)}^{m}}$$

(7) $$x_{jk}^{*}=\sum_{i=1}^{c}u(x_{k},y_{ji})\cdot y_{ji}$$

It should be noted that although the terminating condition of the conventional FCM is met, the final estimation result may not be optimal, because the number of the cluster centroids c and the parameter of the membership degree m are predetermined. Moreover, the clustering performance of the FCM is sensitive to both. Thus, this is the main drawback of the FCM. The way to overcome this shortcoming is to optimize c and m in each condition.

### 3.4. Integrated FCM Imputation Algorithm with GA

The procedure for determining c and m falls into the category of the combinatorial optimization problem. To address this type of problem, the stochastic search algorithm has been proven to be an efficient way [17]. Motivated by the artificial intelligence technique, the genetic algorithm (GA) is effective in solving the combinatorial optimization problem and providing an excellent interface with other algorithms. Thus, in our study, the GA was implemented in the FCM to optimize the membership degree m and the number of cluster centroids c. In particular, the specific procedure of the integrated imputation algorithm combining the FCM and the GA is put forward as follows:

Step 1: (Initialization) Set the upper bound and the lower bound of both the parameter of the membership degree and the number of the cluster centroids, respectively. Set the threshold ε as 0.1. Initialize the number of the cluster centroids and then calculate the membership degree according to Formulas (3) and (4). Determine the parameters of the GA, including the population size, N=20, the number of generations, T=100, the probability of crossover, $P_{c}=0.9$, and the probability of mutation $P_{m}=0.1$.

Step 2: (FCM) Calculate each membership degree in the FCM. Update the new cluster centroid according to Formula (6), and when the optimal results are obtained, estimate the missing values according to Formula (7).

Step 3: (Evaluation) Evaluate the root mean squared errors (RMSEs) between the estimated values and the actual values using Formula (8). $I_{jk}$ is the binary indicator; when it is equal to 1, then this means the value in the *j*^th^ column and the *k*^th^ row of the matrix is missing, and $x_{jk}^{*}$ is the estimated value, while $x_{jk}$ is the actual value; otherwise, when it is equal to 0, this means the value in the *j*^th^ column and the *k*^th^ row of the matrix exists.

(8) $$RMSE=\sqrt{\frac{\sum_{k=1}^{n}\sum_{j=1}^{p}{\left[I_{jk}(x_{jk}-x_{jk}^{*})\right]}^{2}}{\sum_{k=1}^{n}\sum_{j=1}^{p}I_{jk}}}$$

Step 4: (Fitness calculation) Define the fitness function used in the GA to achieve the selection procedure. The specified fitness is calculated as follows:

(9) f=1/RMSE

Step 5: (GA procedure) Complete the procedures of selection, crossover, and mutation, and then output the new population including c and m.

Step 6: (Termination or not) Judge whether the number of iterations reaches the threshold T or not; if not, then return to Step 2; otherwise, output the missing values.

**Figure 4:**
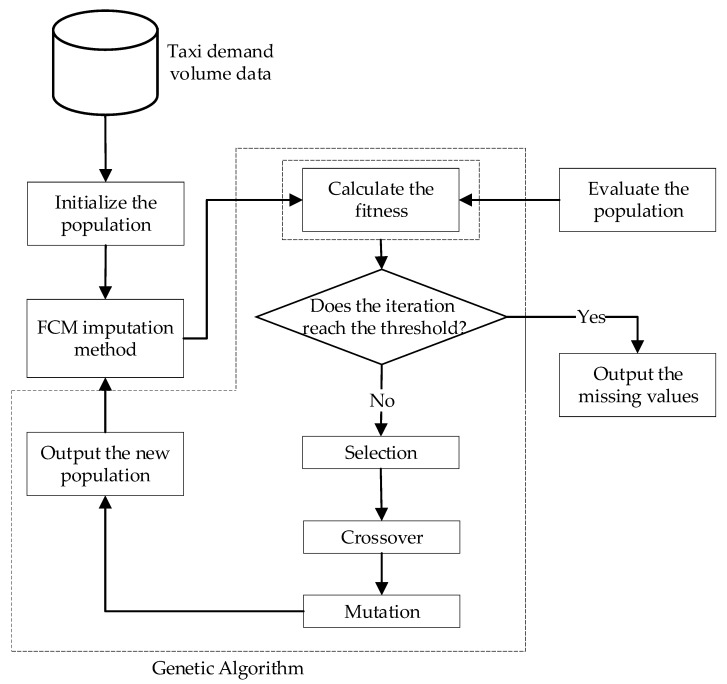
Flowchart of the integrated imputation algorithm.

In summary, the detail of the integrated imputation algorithm is illustrated in Figure 4.

The taxi GPS data in Manhattan, New York City, were utilized to examine the performance of the integrated imputation algorithm. The 24-h taxi GPS data were collected from 14 January to 18 January 2013 (these resources are available in the Supplementary Materials). Moreover, different missing ratios in the datasets were set to evaluate its performance in depth. The detailed results comparisons are put forward in the next section.

## 4. Results and Discussions

### 4.1. Evaluation Criteria of Imputation Performance

Before presenting the comparisons of the results, some optimal parameters obtained by the integrated imputation algorithm are shown in Table 3. In particular, the data were aggregated at four different levels, namely 5, 10, 15, and 20 min, respectively. Correspondingly, the number of time interval was 288, 144, 96, and 72, respectively. Moreover, five kinds of missing ratios, 5%, 10%, 15%, 20%, and 25% are respectively given, and the data were randomly deleted according to the missing ratio.

In this line of research, three evaluation criteria were presented to evaluate the estimation accuracy, which included the root mean squared error (RMSE), the correlation coefficient (*R*), and the relative accuracy (*RA*). Specifically, the RMSE represented the error between the actual values $x_{jk}$ and the estimated values $x_{jk}^{*}$, and the RMSE was calculated by Formula (8). In addition to the error estimation, the R is typically utilized to present the approximation degree between the actual values and the estimated values, and the R was obtained by Formula (10). It should be noted that $\bar{x^{*}}$ is the average value of all missing values, and $\bar{x}$ is the average value of all actual values corresponding to missing values. Meanwhile, the RA reflects the number of estimated results falling within a specified tolerance level, and it was calculated by Formulas (11) and (12). In this study, the specified tolerance level was set as 5% and 10%, respectively. The exact formulas are illustrated as follows:

(10) $$R=\frac{\sum_{k=1}^{n}\sum_{j=1}^{p}I_{jk}(\bar{x^{*}}-x_{jk}^{*})\cdot (\bar{x}-x_{jk})}{\sqrt{\{\sum_{k=1}^{n}\sum_{j=1}^{p}{\left[I_{jk}(\bar{x^{*}}-x_{jk}^{*})\right]}^{2}\}\cdot \{\sum_{k=1}^{n}\sum_{j=1}^{p}{\left[I_{jk}(\bar{x}-x_{jk})\right]}^{2}\}}}$$

(11) $$APE_{jk}=\frac{\left|I_{jk}(x_{jk}-x_{jk}^{*})\right|}{x_{jk}}$$

(12) $$RA=\frac{n_{APE}}{\sum_{k=1}^{n}\sum_{j=1}^{p}I_{jk}}$$

APE is the absolute percentage error, and $n_{APE}$ represents the number of $APE_{jk}$ in the ±5% and ±10% error range.

**Figure 5:**
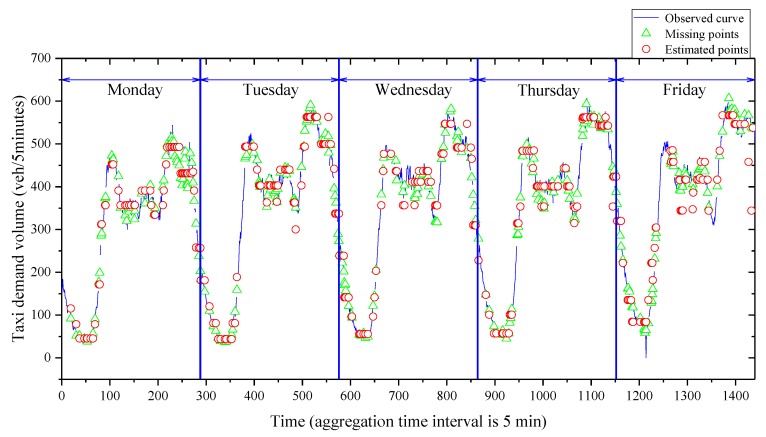
Comparisons between the missing values and the estimated values.

Moreover, based on the condition of 25% missing loss and the five minutes aggregation interval, the actual values of the missing points and the estimated points obtained by the integrated imputation method with the Euclidean distance are shown in Figure 5 to represent their differences.

### 4.2. Comparisons and Analyses of the Results

With the aim of evaluating the integrated imputation algorithm, five imputation methods were compared to demonstrate the effectiveness of the proposed integrated imputation method. The detailed information is proposed as follows:

- The history imputation method. Considering the similarity in data obtained at the same time among different weekdays, in particular, the missing value was replaced by the mean value of the existing values on the same row in the matrix of Table 2 (History). This method also belongs to one of the regression imputation methods.
- The conventional FCM using the Manhattan distance (MDFCM).
- The conventional FCM using the Euclidean distance (EDFCM).
- The integrated FCM–GA using the Manhattan distance (MDFCMGA).
- The integrated FCM–GA using the Euclidean distance (EDFCMGA).

**Figure 6:**
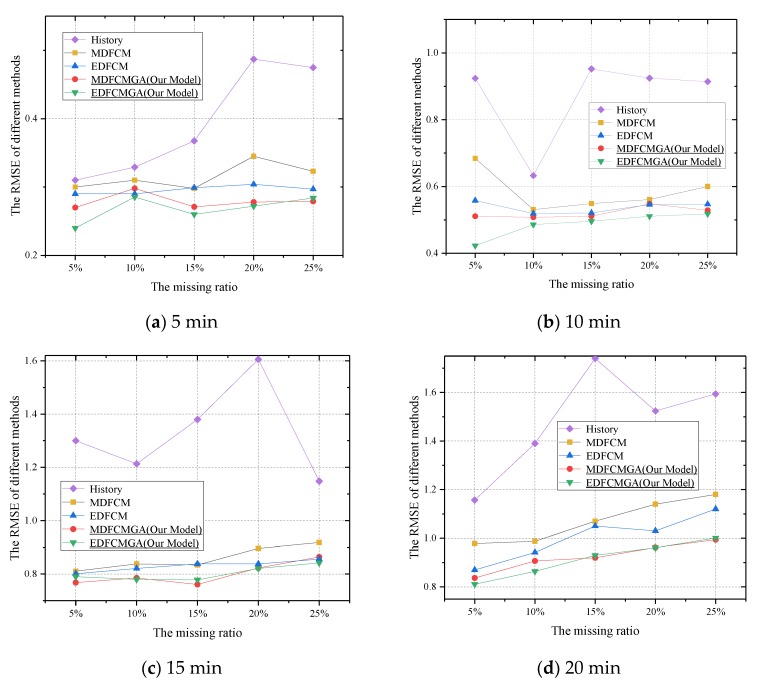
Root mean squared error (RMSE) comparisons under the condition of different data aggregation intervals and different missing ratios.

With respect to the RMSE, as shown in Figure 6a–d, the overall RMSE increased as the missing ratio increased. The reason for this was that when the missing ratio increased with the fixed time interval, the amount of data that could be used in imputation would decrease as well. Thus, the performance of imputation deteriorated. Analogously, as the aggregation interval increased from 5 min to the larger intervals, the overall RMSE would increase simultaneously. The reason for this was that the taxi demand that aggregated from a longer interval was normally greater than that from a shorter interval. Thus, with the same APE, the absolute error between the missing value and the estimated value was larger as well when the time interval was longer. Thus, with a longer interval, the overall RMSE increased as well. In particular, as for the history imputation method, when the aggregation interval was five minutes and the missing ratio was set as 5% (Figure 6a), the gap of the RMSE among the five methods was narrow. However, when the aggregation interval and the missing ratio became larger in Figure 6a, the conventional FCM and the integrated FCM–GA outperformed the history imputation method greatly. The superiority of the FCM and the FCM–GA is also ascertained from Figure 6b–d. Meanwhile, from Figure 6a–d, the RMSE produced by the history imputation method in each figure varied drastically, which indicated that the history imputation method was sensitive to the historical data. In contrast, as for the FCM and the FCM–GA, the RMSE in each figure varied on a small scale, which indicated that the performance of the FCM and the FCM–GA was stable. From Figure 6a–d, we can also see that no matter what the Manhattan or the Euclidean distance was, the RMSE produced by the integrated FCM–GA was smaller than the conventional FCM. The RMSE calculated for the Euclidean distance was smaller than that of the Manhattan distance.

**Figure 7:**
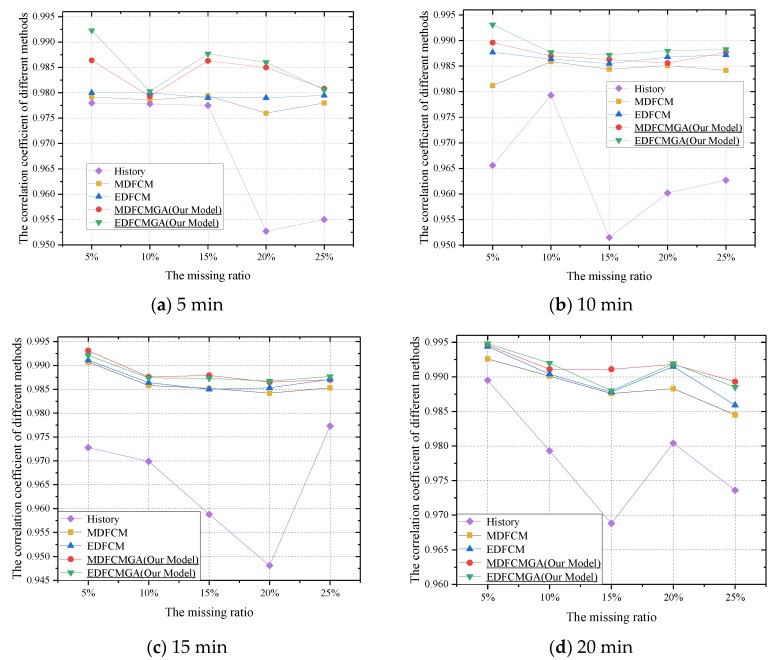
Correlation coefficient comparisons under the condition of different data aggregation intervals and different missing ratios.

It can be seen from Figure 7a–d that as the missing ratio increased, the overall correlation coefficient decreased. This phenomenon was similar to the changes in Figure 6. Evidently, with a higher missing ratio, a decrease in the amount of useful data would also result in a lower correlation coefficient. As seen in Figure 7a, when the aggregation interval was five minutes and the missing ratio were 5%, 10%, and 15%, respectively, the correlation coefficients of the MDFCM, the EDFCM, and the history imputation method were similar. However, when the aggregation interval and the missing ratio became larger in Figure 7a, the conventional FCM and the FCM–GA outperformed the history imputation method substantially. The effectiveness of the FCM and the FCM–GA is also shown in Figure 7b–d. Meanwhile, as seen in Figure 7a–d, the correlation coefficient obtained from the FCM and the FCM–GA remained stable from 0.98 to one, which demonstrated the fact that the missing values had a close relationship with the existing values. Moreover, this phenomenon indicated that missing values could be estimated by using the existing values, which was coherent with our proposed assumption. The integrated imputation algorithm based on the FCM and the GA with the Manhattan and the Euclidean distance also resulted in the two highest values in Figure 7a–d.

**Figure 8:**
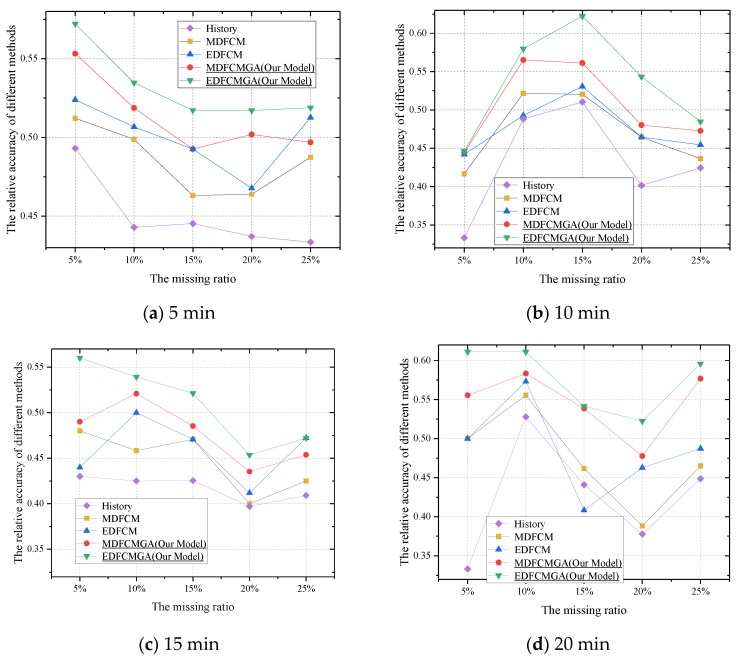
Relative accuracy (RA) comparisons under the condition of different data aggregation intervals, different missing ratios, and the ±5% tolerance error range.

**Figure 9:**
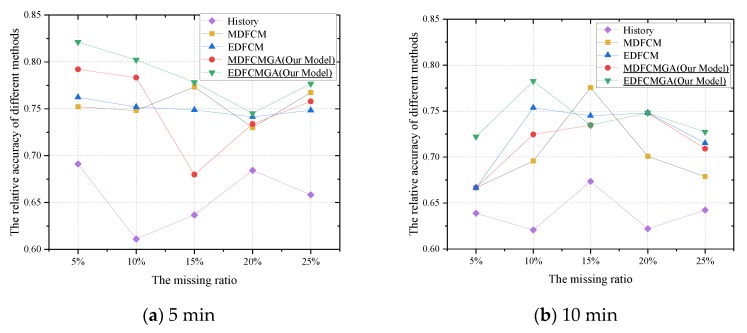

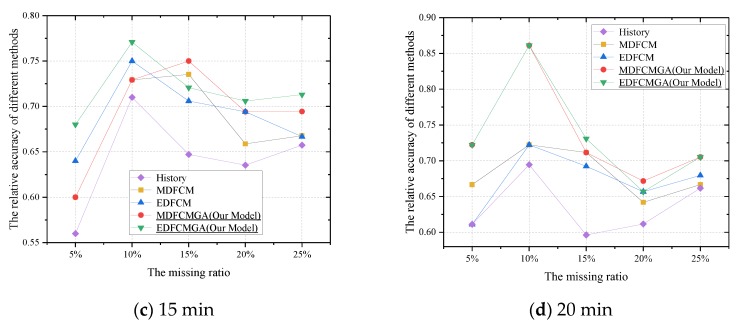
RA comparisons under the condition of different data aggregation intervals, different missing ratios, and the ±10% tolerance error range.

To reflect the superiority of the integrated imputation method in depth, the RA was calculated according to two scenarios, including ±5% and ±10%. From Figure 8a–d, when the threshold was set strictly as ±5%, the integrated imputation method outperformed the other methods significantly. Meanwhile, the performance of the integrated imputation method in Figure 8a–d was consistently stable. Moreover, the RA obtained by the FCM–GA with the Euclidean distance type had the highest value in Figure 8a–d. As seen in Figure 9a–d, when the threshold was set less strictly as ±10%, the integrated imputation method still outperformed the other methods. On the other hand, although the performance of the conventional FCM in Figure 9b with a 15% missing ratio was remarkably enhanced, its imputation performance was still unstable under other scenarios. In other words, the conventional FCM was sensitive to the time interval and missing ratio. With respect to the integrated imputation method, the integrated imputation method was less sensitive to the time interval and missing ratio. Therefore, although the phenomenon from Figure 9 indicated that the conventional FCM was a possible substitute when the condition was relaxed, and the integrated imputation method was still a stable and reliable choice for estimating missing values.

Another interesting phenomenon was also derived from the results presented in Figure 8 and Figure 9. When the threshold was strictly set as ±5%, the average relative accuracy (RAs) obtained by the EDFCMGA were, respectively, 0.532, 0.535, 0.509, and 0.576 under the 5, 10, 15, and 20 min aggregation time intervals. Thus, the best aggregation time interval should be set as 20 min under this scenario. In addition, when the threshold was less strictly set as ±10%, the average RAs obtained by the EDFCMGA were, respectively, 0.785, 0.742, 0.718, and 0.735 under the 5, 10, 15, and 20 min aggregation time intervals. Thus, the best aggregation time interval should be set as five minutes under this scenario. In general, a longer aggregation time interval would remove randomness, which is typically viewed as a certain smoother filter. However, a longer aggregation time interval may conceal available variation information as well. In fact, the accuracy of imputation is highly associated with the detailed information in a dataset. Thus, how to determine the aggregation time interval in the imputation is another hot topic [47,48].

**Figure 10:**
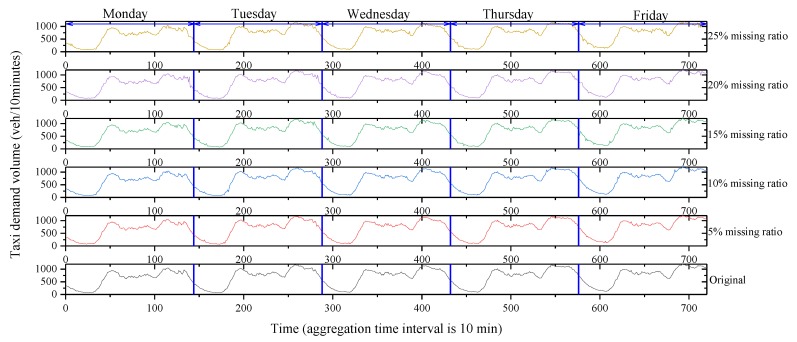
Time series comparisons with different missing ratios.

To reinforce our interpretation of the superiority of the proposed integrated FCM imputation method, an example that covers the time series of observed curve and estimated values is presented in Figure 10. In Figure 10, five scenarios with different missing ratios are respectively given. By comparing the estimated time series with the original time series, we found that the estimated time series under different scenarios was close to the original one. Thus, in terms of different missing ratios, our proposed integrated imputation method could be applicable to estimating missing values in the time series.

In Table 4, the average computation time in seconds is described. It should be noted that all experiments were conducted on a personal computer with an Intel Core i5-6200U (Bayan Lepas, Malaysia) and 12 GB RAM. Meanwhile, ten repeated tests of each scenario were carried out, and the average computation time of each scenario was obtained. From Figure 4, it can be seen that the FCM-GA contained not only a complete FCM computation procedure, but also parameter updates. Theoretically, the computation time of the FCM-GA should be higher than that of the FCM. However, as seen in Table 4, the experimental results were coherent with the theoretical analyses. As a consequence, the better imputation accuracy of the FCM-GA is also inferred from Figure 6, Figure 7, Figure 8 and Figure 9. In other words, we compromised on computation time to achieve better imputation performance. Better imputation accuracy indeed has a higher priority than computation time in dealing with missing value imputation issues [49].

In summary, when the aggregation interval and missing ratio were smaller simultaneously, the history imputation method was an alternative approach to estimate the missing values. The advantage of the history imputation method lay in its simplicity. However, when the aggregation interval and the missing ratio became larger, other complicated imputation methods with better performance should be utilized to replace the history imputation method. The conventional FCM would be another valid candidate choice for estimating missing values. However, the main drawback of the conventional FCM is that its parameters are not determined scientifically. Considering this, the proposed integrated imputation method combining the FCM and the GA could be utilized to replace missing values efficiently. From Figure 6, Figure 7, Figure 8 and Figure 9, the results indicate that the integrated imputation method outperformed the conventional FCM remarkably.

## 5. Conclusions

Due to sensor or software failures, missing traffic flow data occur typically in Intelligent Transportation System (ITS) datasets. How to handle these missing values has become a fundamental issue to guarantee data quality.

In this study, an integrated FCM imputation method based on the GA was proposed to estimate the missing values in the datasets. Specifically, the GA dealt with two critical parameters utilized in the FCM, and the FCM addressed the missing values based on the clustering technique. Within the specific framework of the integrated imputation method, a matrix-based data structure was utilized to better reflect the data similarity. The effectiveness and the superiority of the integrated imputation method were revealed based on the experimental test with different scenarios.

Although the integrated imputation method used in this paper could be applicable to estimating the missing values efficiently, some limitations existed. Firstly, in our study, only the taxi GPS dataset was utilized to estimate the missing values. In fact, traffic conditions and weather information may also influence the estimation results. Secondly, we assumed that the missing pattern only belonged to the MPR; however, the realistic situation is more complex. Thus, in future work, to estimate missing values more accurately, multiple dimensional datasets will be utilized. Meanwhile, some stochastic processes (e.g., the Dirichlet process) could be utilized to address the independence of the variables. Moreover, based on the estimated values and existing values, an extension of how to achieve the prediction task is another interesting issue that warrants further research.

## Figures and Tables

**Table 1:** Samples of taxi GPS data.

PT	DT	PLO	PLA	DLO	DLA	TD	TDI
2013-1-13 4:36	2013-1-13 4:46	−73.9969	40.72006	−73.9935	40.69304	600	3.12
2013-1-13 4:37	2013-1-13 4:48	−74.0003	40.73007	−73.9874	40.76841	660	3.39
2013-1-13 4:41	2013-1-13 4:45	−73.9973	40.72098	−74.0004	40.73238	240	1.16
…	…	…	…	…	…	…	…

PT means pick-up time; DT means drop-off time; PLO means pick-up longitude; PLA means pick-up latitude; DLO means drop-off longitude; DLA means drop-off latitude; TD means trip duration (seconds); TDI means trip distance (km).

**Table 2:** Taxi demand volume on weekdays. The “question marks” in Table 2 represent the missing values.

	Monday	Tuesday	Wednesday	Thursday	Friday
0:00:00–0:05:00	158	?	?	249	341
0:05:00–0:10:00	184	200	254	?	?
0:10:00–0:15:00	163	200	263	248	341
…	…	…	…	…	…
7:30:00–7:35:00	?	405	421	417	407
7:35:00–7:40:00	399	444	?	455	435
7:40:00–7:45:00	429	?	?	?	468
…	…	…	…	…	…
23:45:00–23:50:00	240	?	286	395	549
23:50:00–23:55:00	205	281	284	398	542
23:55:00–0:00:00	?	?	282	?	509

**Table 3:** Optimized parameters obtained by the integrated imputation method.

Time Interval (min)	Manhattan Distance		Euclidean Distance	
	*c*	*m*	*c*	*m*
5 min	12	1.1083	14	1.1936
10 min	12	1.1123	14	1.1183
15 min	13	1.1016	12	1.1145
20 min	8	1.1426	9	1.1211

**Table 4:** Average computation time of FCM and FCM-GA (in seconds).

Time Interval (min)	Missing Ratio	FCM	FCMGA
5 min	5%	32.90	258.30
	10%	32.99	259.40
	15%	33.01	260.10
	20%	33.18	262.30
	25%	34.33	264.87
10 min	5%	12.59	221.90
	10%	12.61	224.10
	15%	14.22	224.96
	20%	14.90	225.64
	25%	17.39	228.22
15 min	5%	9.08	168.31
	10%	9.39	169.32
	15%	9.67	170.20
	20%	11.87	171.99
	25%	11.90	172.40
20 min	5%	8.14	89.20
	10%	8.66	91.20
	15%	8.80	91.88
	20%	9.22	92.51
	25%	9.80	93.91

## Supplementary Materials

The taxi GPS data utilized in this paper is also available online at https://www1.nyc.gov/site/tlc/about/tlc-trip-record-data.page.
